# Online Food Choices: When Do “Recommended By” Labels Work?

**DOI:** 10.3390/foods13060928

**Published:** 2024-03-19

**Authors:** Daniele Catarci, Lea Laasner Vogt, Ester Reijnen

**Affiliations:** School of Applied Psychology, Zurich University of Applied Sciences, 8005 Zurich, Switzerland; lea.laasner@zhaw.ch (L.L.V.); ester.reijnen@zhaw.ch (E.R.)

**Keywords:** recommendation, food choices, price, position

## Abstract

Understanding digital menu choices in limited-option environments, such as university cafés, is crucial for promoting healthier and more sustainable food choices. We are, therefore, looking at two possible interventions or nudges—recommendation and position—and how they interact with, for example, price. In the first smartphone-based study (*N* = 517), participants were presented with two menu options, while the factors “recommendation”, “position”, and “price” were manipulated. We only found effects in relation to the choice of the more popular menu option. Specifically, when the popular meal was the expensive option, the recommendation had a negative effect on choice, but not when the popular meal was the cheaper option. The aim of the second smartphone-based study (*N* = 916) was to shed more light on the role of popularity or personal preference in relation to recommendations. We manipulated the differences in personal preference (small or large) using a ranking task presented before the menu choice. In Study 2, the interaction effect between recommendation and price for the more popular menu option could not be replicated. Instead, we found that the greater the difference in preference, the less pronounced the price effect was. Overall, some effects of the recommendations have been identified, but further research is needed to clarify the exact circumstances under which they arise.

## 1. Introduction

On a Friday evening out with friends, you may find it difficult to choose from the restaurant menu. Will you have the quinoa power pot? Or maybe the beany shepherd’s pie? It is likely that you will choose the menu option that *satisfies* you the most at that moment (i.e., in terms of *taste*). However, is that the best meal choice when it comes to tackling broader societal challenges such as obesity? In Switzerland alone, 42% of the population is overweight; 11% is obese (data from 2017) [1]. Choosing a *low-calorie*, balanced, and nutritious meal is crucial for your health and helps to prevent noncommunicable diseases (NCDs) such as cardiovascular diseases (e.g., heart attacks), cancers, and diabetes [2,3,4,5,6]. If the chosen menu option is also more *regional* and *plant-based*, it also contributes to tackling another global issue, namely reducing the carbon footprint associated with food [7,8,9,10].

A more plant-based diet could be achieved by reducing meat consumption or replacing meat with plant-based meat alternatives (PBMAs) [11]. However, one of the main barriers to the latter is that people *enjoy eating meat* [12]. Accordingly, frequent meat eaters rate PBMAs anything but positively and are less likely to opt for plant-based substitutes [11] (see also [13] for a negative correlation between red meat consumption and PBMAs). Furthermore, only people with certain socio-demographic characteristics, for example, people with *high incomes* or *women*, currently respond to PBMAs. The fact that people who show a general interest in health [13] also respond to PBMAs could (in addition to environmental awareness) serve as a clue for the promotion of these products [14]. However, it is important to note that PBMAs, which are often highly processed, should not automatically be considered healthier than conventional meat dishes [11]. Without digressing into the ongoing debate about what a perfectly healthy, eco-conscious diet should look like, in this manuscript, we look at how people can be persuaded to choose, for example, the healthier option on the restaurant menu.

However, making “good or better” menu decisions is anything but easy. According to the standard classical economic model of human decision-making, this requires not only that you be *aware* of all the relevant information (e.g., sugar content) but also that you are able to weight it up correctly (e.g., sugar is twice as harmful as fat). However, this requires some kind of *cognitive effort* (or the use of Kahneman and Tversky’s System 2 [15,16,17,18], which we tend to *avoid*. Last but not least, you must be self-controlled and not let yourself be led by your feelings (e.g., “Hmmm, I’ve had a bad day, so I’m craving the pie tonight”). In view of all these factors that make decision-making a difficult process, it is, therefore, not surprising that around 95% of our daily decisions (including food-related decisions) tend to be gut decisions or, to put it scientifically, *automatic* decisions (or System 1 decisions [15]). Besides the fact that automatic decisions are based on processes opposite to the ones underlying the decisions mentioned above (e.g., unconscious versus conscious, fast versus slow, stimulus-based versus goal-oriented, implicit versus explicit, etc.; see Kahneman, 2011), in our context, it is particularly important that automatic decisions are based on *heuristics* (e.g., “chocolate is unhealthy”), that is, rules-of-thumb which most of the time lead to the correct choice, but not always.

Importantly, these heuristics are sensitive to *contextual* or *environmental* cues, such as color. Since the brain prefers multicolor to monochrome, Paakki et al. (2019) conclude: “using intensive colors and stimulating color combinations is one potential way to tempt consumers to choose and consume more vegetables” [19] (p. 81). See [20], how using more colors in M&M’s leads consumers to consume more of this unhealthy chocolate. The realization that cues such as color or their manipulation can be used to steer human decisions in a certain direction has been extensively researched in recent years as part of the so-called *nudging approach*, including in the food sector, such as the restaurant and hospitality industry. For example, using smaller plates at a buffet reduces the amount of food consumed and, therefore, reduces food waste [21]. A nudge, therefore, “influences choices in a way that will make choosers better off” [22] (p. 5) by changing the decision-making environment (e.g., plate size). It is crucial that *better off* is to be interpreted as: “judged by themselves”—for instance, someone who wishes to lose weight may prefer to eat less. In addition, in the nudging approach—in order to ensure freedom of choice—one must neither remove options, such as unhealthy menu options (e.g., remove the tiramisu from the menu), nor overprice menu options (e.g., make the tiramisu extremely expensive).

An overview of nudges that are used and researched in the food sector can be found in the meta-analysis by Cadario and Chandon (2020) [23]. Therein, the authors divide nudges into the following three categories: cognitive, affective, and behavioral. A nudge whose processing requires little attention is classified as *cognitive*. This includes *labels* such as “heart healthy”, which quickly and easily communicate which products are healthy. This also includes all measures that improve the visibility of products, for example, placing healthier products at eye level on the shelf or healthier meal options at the top of the menu. If, on the other hand, a dish is described as “Aunt Anna’s apple pie” (instead of apple pie), an *affective* nudge is used. Finally, the above-mentioned example of using smaller plates at the buffet is a *behavioral* nudge. According to the meta-analysis, behavioral nudges are the most effective, followed by affective nudges, with cognitive nudges at the lower end of the success spectrum. Overall, nudges offer a promising tool for guiding consumers’ food choices in a simple and cost-effective way towards healthier choices without restricting their freedom of choice [24]. However, the responsibility to act in an ethically and morally sound way must not be ignored.

The research question investigated in this paper is: How can restaurants or *university cafeterias* in particular use *nudges* to promote certain menu options? It should be noted that a unique feature of university cafeterias is that their menu selection is usually limited to two or a maximum of three options. Furthermore, as the transition to digital menus and, thus, the increasing use of smartphones for food selection is a noticeable reality in this environment, we are particularly interested in nudges that can be implemented “digitally”. Although the digital presentation of menus is becoming more and more fashionable, there is little to no research on the subject [25].

Due to the broader literature on nudging, one particularly relevant nudge to test for in our context is the “menu position”. In recent years, numerous studies have tried to find out how the position of menu options influences their choice. The influence of factors such as the arrangement of the menu options (horizontal or vertical), the variability of the available meals (e.g., only desserts), the number of menus displayed (few or many), etc., have all been investigated. In 2015, Bar–Hillel announced that the mystery of which position was the “best” in terms of preferred product selection had been solved. She claimed that if the consumer has to choose between vertically arranged (menu) options that are different from each other (e.g., curry and spaghetti) and, therefore, require cognitive, effortful processing, an “*edge advantage*” effect should be observed [26]. This means that menu options located at the top or bottom of the menu are preferentially selected [27] (see [28] on tacos). However, there are also findings that contradict these results. For example, the study by Pinger et al. (2016) with “real data” from a specialty restaurant shows the opposite effect, the so-called “*edge aversion or compromise*” effect [29]. Menu options placed in the middle are preferentially selected (probably due to the rule of thumb that the middle options contain the most popular meals [18]). Moreover, other studies show no menu position effect at all (e.g., [30]; [31], see review by Ozdemir and Caliskan, 2015 [32]). Finally, to our knowledge, there are no studies that investigate what kind of positional effect, if any, can be expected if only two options are offered, as is common in university canteens.

We also investigate the effects of the cognitive nudge of recommendation, such as the “chef’s suggestion”. Recommendations are considered helpful, as it is difficult to evaluate dining experiences and customers are, therefore, reliant on external information [33]. Although recommendations are widely used in the catering industry, there is little research on their effectiveness, especially in binary food choices. One of the few exceptions is the study by Bookwala et al. (2022) [34], in which the participants could choose between two muffins (e.g., A versus B). One of the muffins contained a recommendation either in the form of an informative label (e.g., Muffin A: “baker’s choice” or Muffin B: “most popular”) or in the form of a generic label (Muffin A or B with a “thumbs up”). In addition to these four conditions or groups, there was a fifth group, the so-called classic control group without any recommendation. Note that the “baker’s choice” label was placed only on, for example, Muffin A but not on Muffin B (the opposite was true for the “most popular” label). This was because the same muffin could not be both the baker’s choice and the most popular. The authors found that, compared to the control group, an effect of the recommendation on choice was only observed when the label was on the *most frequently* chosen product, and in this respect, a significant effect was only observed when the recommended label was an informative one (here: the “most popular” label; see [35] on why the “chef’s choice” label may not work). However, on closer examination of their results, the authors concluded that the results may be due to cultural differences. While non-natives responded positively to the label “most popular”, locals did not seem to respond to it at all. Similarly, Yang and Mattila (2020) identified a cultural component in recommendations. While Indians seemed to prefer recommendations based on popularity, Americans seemed to prefer recommendations based on expertise [36] (see [34] for no effect of expertise on natives). However, the study by Yang and Mattila did not include a classic control group, so it is unclear whether and how the recommendation nudge influenced choice.

In summary, with this paper, we hope to contribute towards closing some research gaps. Firstly, we want to investigate whether—due to a *limited choice* of two menu options in an online setting—the position of the menu option influences its choice (and if so, how). Secondly, we want to investigate how *recommendations* (in this case general recommendations) influence menu choice in interaction with position. In this regard, we also add the often-forgotten classical control condition. Last but not least, we want to investigate the influence of menu prices in interaction with position and recommendation. In doing so, we do not consider the price of the meal as a nudge but as an integral part of the meal itself. We include price because Andaleeb and Caskey (2007) found (using a questionnaire survey) that price is decisive in terms of customer satisfaction in college cafeterias [37], (see [38] on the importance of price when buying tomatoes, for example).

## 2. Study 1

### 2.1. Materials and Methods

#### 2.1.1. Participants

In total, 517 students between the ages of 18 and 45 (*M* = 24.0, *SD* = 3.96; of whom 63.4% are female) from the ZHAW University of Applied Sciences participated in this smartphone-based online study. The students were recruited via the universities’ internal mailing lists and, thus, form an ad-hoc sample. Regarding diet, 377 participants (72.9%) reported that they did not follow a specific diet, 113 participants (21.9%) said that they followed a vegetarian diet, and 27 (5.2%) said they followed a vegan diet. As a reward, participants could either enter a raffle for four CHF 50 gift certificates (88.2%), or students at the School of Applied Psychology (13.0%) could receive course credit instead (which 52.2% of them did). All participants provided their informed consent (according to the guidelines of the ZHAW, there are no ethical objections to this study).

#### 2.1.2. Stimulus Material, Procedure, and Design

**Figure 1:**
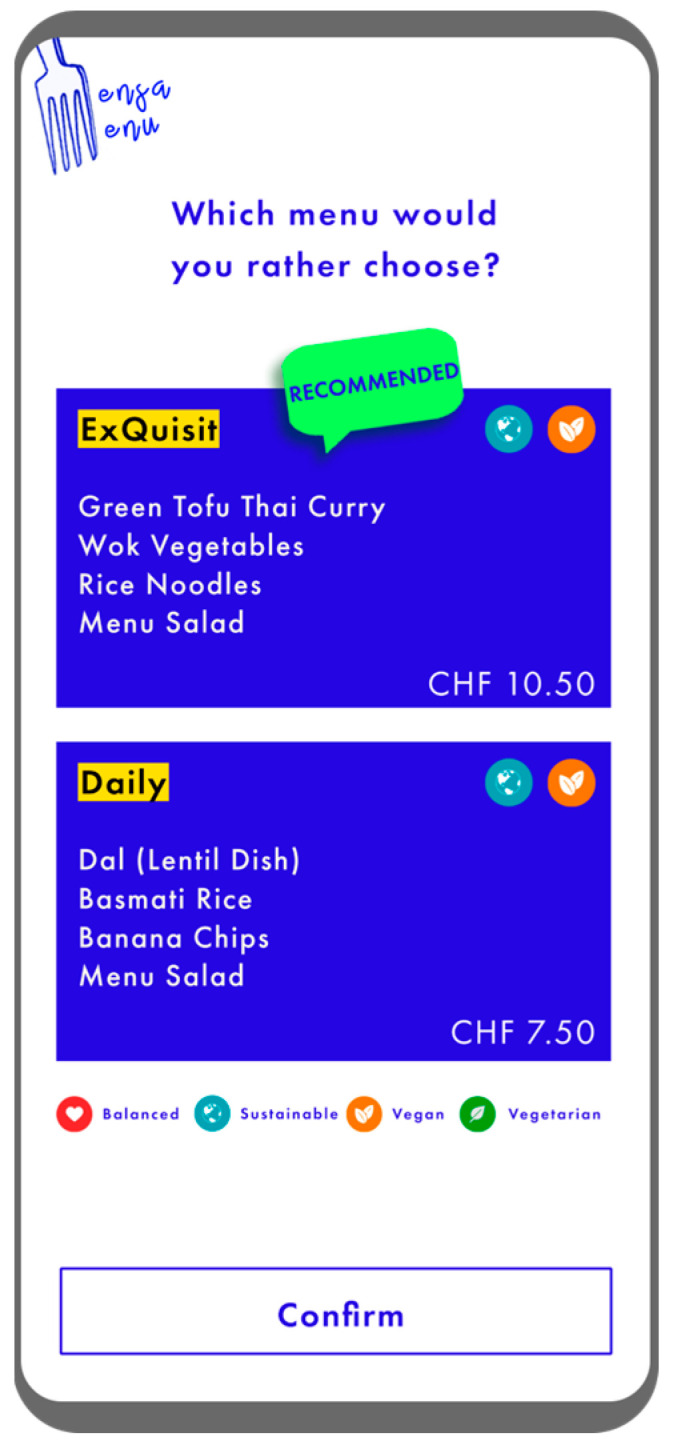
Stimulus material (translated into English). Note that the expensive menu was always labeled as “ExQuisit”, the cheaper menu as “Daily”.

Participants’ task—in an online survey programmed with Unipark (version 22.1; 31 May 2022)—was to make a binary choice between two vertically arranged menu options: a curry and a dal (see Figure 1). Depending on the condition (in total 12) to which the participants were randomly assigned to, for example, the curry was positioned at the top (factor curry position: top, bottom) and was the expensive menu option (factor curry price: cheap, expensive). In addition, a meal could feature a recommendation (factor menu recommendation: none, curry recommended, dal recommended).

After participants made their choice, they had to rate a number of factors (e.g., price, balance, variety, taste, popularity, recommendation, sustainability, health, etc.; summarized under “menu choice relevant factors”) in terms of their importance for menu selection in the cafeteria on a five-point-scale (1 = not at all important, 5 = very important). Finally, before we collected participants’ demographic data (e.g., age, gender), participants were presented with the recommendation banner and asked to state their opinion on the meaning and purpose of that banner.

### 2.2. Results

Apart from 22 participants who took more than 20 min to complete the study and were, therefore, removed from the analysis (4.1% of the total), all data collected were included in the analysis (except for data from participants who dropped out of the study). All analyses were conducted with R (version 4.2.3; 15 March 2023).

**Figure 2:**
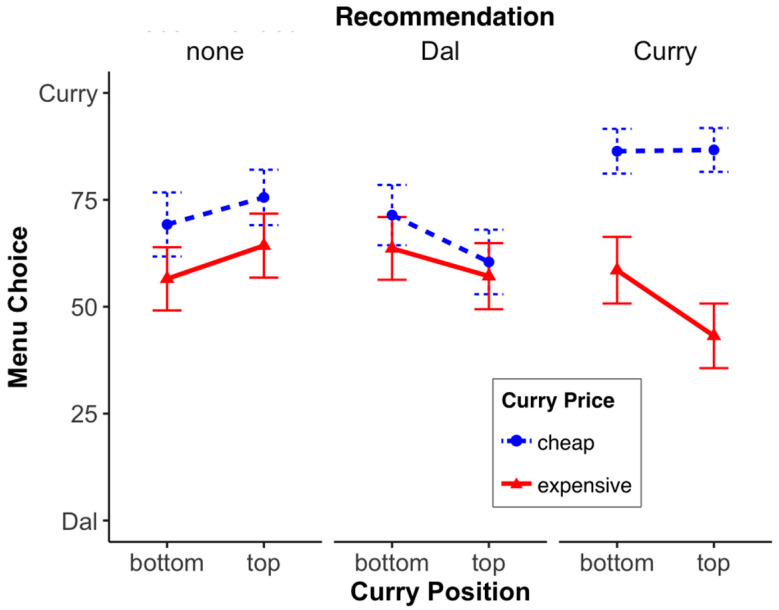
Menu choice (0 = everyone chooses the dal menu, 100 = everyone chooses the curry menu). For the different factor (recommended, curry position, curry price) combinations, the error bars represent the standard error.

**Figure 3:**
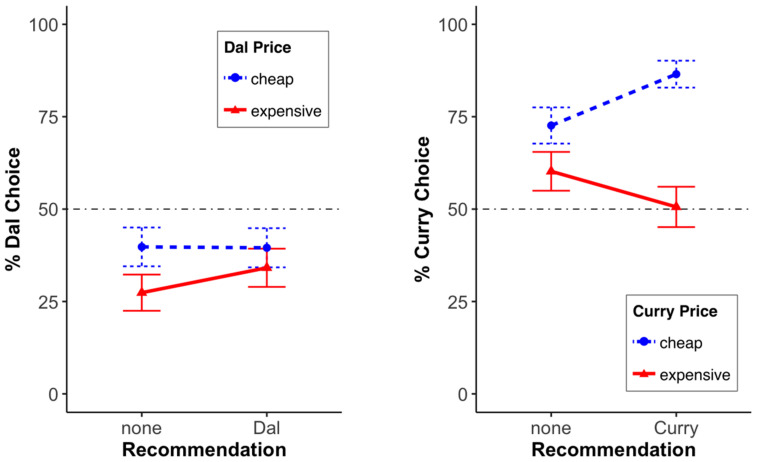
Impact of the recommendation on the choice of the dal option (**left**) and the curry option (**right**). The error bars represent the standard error.

*Menu choice:* We conducted a 3 (recommendation: none, curry, dal) × 2 (curry position or in short “position”: bottom, top) × 2 (curry price or in short “price”: cheap, expensive) between-subjects probit model (including an ANOVA) on menu choice (binary variable). While the main effects of recommendation, *χ*^2^ (2, *N* = 517) = 1.48, *p* = 0.477, and position, *χ*^2^ (1, *N* = 517) = 0.52, *p* = 0.473, were not significant, there was a significant main effect of price, *χ*^2^ (1, *N* = 517) = 18.96, *p* < 0.001. In other words, curry was chosen more often when it was cheap than when it was expensive. With respect to the two-way interactions, we found that the recommendation × position interaction, *χ*^2^ (2, *N* = 517) = 3.00, *p* = 0.223, and position × price, *χ*^2^ (1, *N* = 517) = 0.08, *p* = 0.774, were not significant, but the *recommendation* × *price* was significant, *χ*^2^ (2, *N* = 517) = 11.60, *p* < 0.01. Finally, the three-way interaction of recommendation × position × price was not significant, *χ*^2^ (2, *N* = 517) = 0.90, *p* = 0.639 (see Figure 2 or Figure 3; both display the same data, but in a different way).

To find out what caused the significant (menu) recommendation × price interaction, we ran three separate analyses (one for each level of recommendation). We found that while price had no significant effect in the conditions where no meal or the dal meal was recommended (none: *χ^2^* (1, *n* = 172) = 2.78, *p* = 0.095; dal: *χ^2^* (1, *n* = 171) = 0.58, *p* = 0.446), price did have an effect when the curry meal was recommended. Hence, when the (overall) more popular meal was recommended (chosen in 66% of the cases), the cheap curry was chosen more often: *χ^2^* (1, *n* = 174) = 27.24, *p* < 0.001. None of the position effects were significant (all *χ*^2^’s ≤ 1.41; all *p*’s ≥ 0.236). In summary, if the more popular menu option, here, the curry, was cheap, its recommendation significantly increased its choice.

*Banner interpretation*. The question arises as to whether the significant recommendation × price interaction result above is caused by the way participants interpret the “recommended” banner? To answer this question, the verbal statements of the participants were divided into the following three categories: nudge, marketing, or neutral (see [39] for the used categories). If “recommended” was perceived as benevolent in terms of, for example, taste, the statement was categorized as a nudge (e.g., “A menu is likely to be recommended because of value for money, eco-friendliness, and taste”). On the other hand, if “recommended” was perceived as a cafeteria strategy to increase revenue (e.g., “It should encourage customers to buy this menu. Maybe it will bring more profit with less effort”), the statement was classified as marketing. If participants were undecided regarding the intent of the recommendation, their statement was classified as neutral. Overall, 43% of the participants interpreted the “recommended” as a nudge, 20% as a marketing strategy, and 37% as neutral.

**Figure 4:**
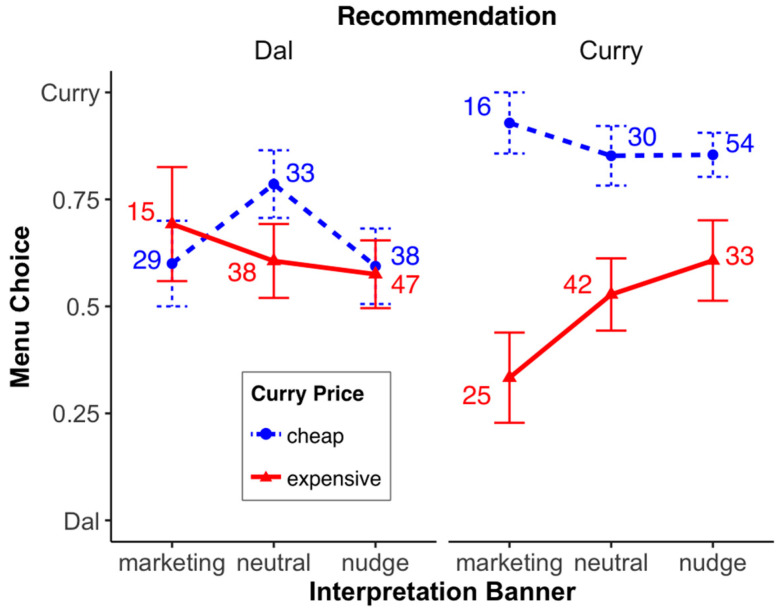
Menu choice (0 = everyone chooses the dal, 100 = everyone chooses the curry) for the different factor combinations (recommended, curry price, recommendation interpretation). Error bars denote the standard error of the means. The numbers above the data points show the percentage of participants who—within a “recommended × curry price” combination—considered the banner as marketing strategy, as neutral, or as nudge. For example, when the curry was recommended and cheap, 16% of the participants considered the banner as a marketing strategy, 30% as neutral, and 54% as a nudge.

For the analysis, we ran a probit model (including an ANOVA) with the independent variables price, recommendation (without the level “none”), and banner interpretation on menu choice (binary variable). Again, we only found a significant main effect of price, *χ*^2^ (1, *n* = 345) = 15.49, *p* < 0.001, and a significant recommendation × price interaction, *χ*^2^ (1, *n* = 345) = 9.25, *p* < 0.01 (see Figure 4; see footnote for interpretation of the numbers; all other main effects or interactions (2- or 3-fold) were not significant: all *χ*^2^′s ≤ 4.08, all *p*’s ≥ 0.130).

**Figure 5:**
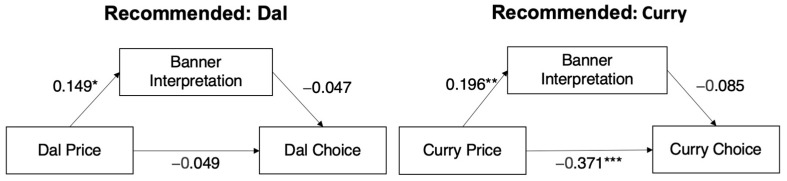
Conditional mediation model. Coefficients represent standardized path coefficients. Note: *** *p* < 0.001; ** *p* < 0.01; * *p* < 0.05. The indirect effect from price to menu choice via banner interpretation was non-significant (*b* = 0.032, *p* = 0.211).

In view of the results, the banner interpretation does not act as a moderator. But could it have acted as a *mediator*? To test this, we performed a conditional mediation analysis with the Lavaan package. As can be seen in Figure 5, price only had a significant effect on menu choice when the curry (but not the dal) was recommended. Moreover, while price had a significant effect on the interpretation of the banner (for dal and curry), the interpretation of the banner had no effect on the choice of curry (with a standardized effect of 0.032 and *z* = 1.249, *p* = 0.211) or on the choice of dal (with a standardized effect of 0.002 and *z* = 0.469 (*p* = 0.639).

**Figure 6:**
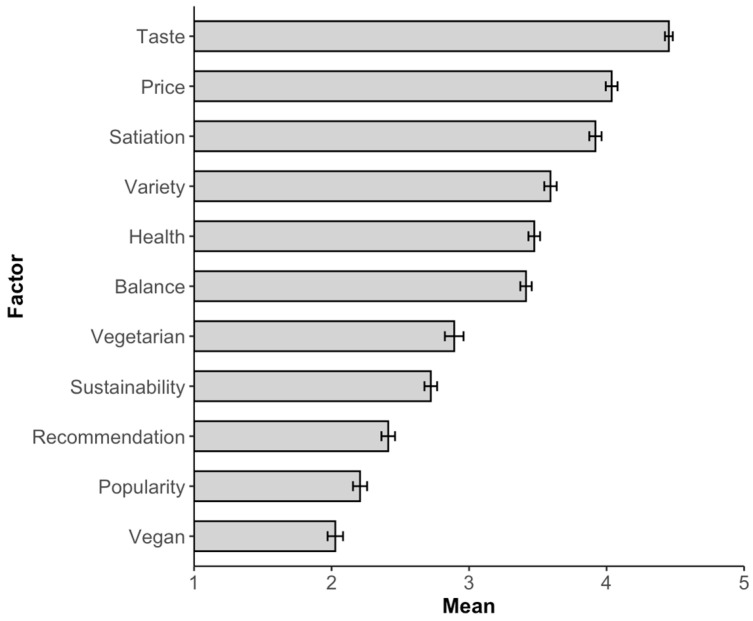
The relative importance of various factors influencing participants’ menu choices in the cafeteria (1 = not at all important to 5 = very important). The error bars represent the standard error.

*Menu choice relevant factors (descriptive).* According to the self-reports of the participants, the most important factors for the menu choice in the cafeteria are taste, followed by price and satiety. In contrast, recommendation, popularity, and whether the menu is vegan (see Figure 6) seem to be factors that play a less important role.

### 2.3. Discussion

Study 1 showed that the recommendation had an effect when it was placed on the more *popular*, cheaper menu (see [21] for similar results). It did not matter whether the banner was interpreted as a nudge or a marketing strategy. In contrast to the results of Smits (2023) [40], the recommendation did not, per se, lead to an increase in the choice of the more popular menu option, but only if the popular menu option was also cheap (Smith did not manipulate the price variable). Considering that participants considered taste to be the most important factor when choosing a meal, we might have observed a stronger effect of the recommendation if it had been less general and more specific to taste (e.g., “tastiest”). However, it is questionable whether a factor that is explicitly regarded as “(personally) more important” when choosing from a menu (e.g., taste over health) also carries more weight when actually choosing from a menu. For example, Zanstra et al. (2017) showed that their participants were more likely to choose a soup that contained a health label (i.e., “reduced salt”) than a taste label [41]. Due to our ranking of factors, the opposite effect should have been observed. Overall, in Study 1, the position of the menu options did not play a role.

## 3. Study 2

In Study 2, we will take a closer look at the role of *popularity* in relation to the impact of recommendations. We thereby assume that popularity is equivalent to people’s *personal preference*, in our case, for a particular menu option. What do we know about its effect? For example, Reijnen et al. (2019) have shown that if you prefer the cuisine of the restaurant you eat at (e.g., Mexican or Chinese), meals in the middle of the menu are preferred [42]. This so-called *edge aversion* effect can be explained by the fact that people know that the most popular menus are usually displayed in the middle. If, on the other hand, you do not prefer the cuisine of the restaurant, you subjectively perceive all menus as similar, which not only reduces the cognitive effort involved in choosing, but also eliminates the effect of edge aversion. Personal preferences (here: the cuisine of the restaurant), therefore, seem to moderate position effects and, thus, one’s choice. Similarly, Gidloef et al. (2017) showed that preferred (shopping) items have a higher probability of being noticed (and selected accordingly) [43]. However, they found that preference (a so-called internal factor) is not independent of external factors such as saliency (i.e., how well a product stands out from the surrounding products). Therefore, they state that “consumers can take advantage of salience when it works in their favor” (p. 36). Hence, salient products are more often looked at if they match the customers’ preference. However, it is still unclear whether internal and external factors always support each other or whether they can also work against each other [44]. Thus, if we replace popularity with personal preference, we should observe the same effect as in Study 1; however, the effect should become smaller as the distance between preferred and non-preferred menu options increases (see voting behavior as an explanation in the discussion section).

### 3.1. Materials and Methods

#### 3.1.1. Participants

In total, 916 students between the ages of 18 and 55 (*M* = 24.4, *SD* = 4.73; of whom 71.2% female) from the ZHAW University of Applied Sciences participated in this smartphone-based online study (the sampling method is the same as in Study 1). Regarding diet, 677 participants (73.9%) reported that they did not follow a specific diet, 191 participants (20.9%) said they followed a vegetarian diet, and 48 (5.2%) said they followed a vegan diet. As a reward, participants could either enter a drawing for six CHF 50 gift certificates (92.7%), or School of Applied Psychology students (8.7%) could receive course credit instead (which 28.8% of them did). All participants provided their informed consent (according to the guidelines of the ZHAW, there are no ethical objections to this study).

#### 3.1.2. Stimulus Material, Procedure, and Design

**Figure 7:**
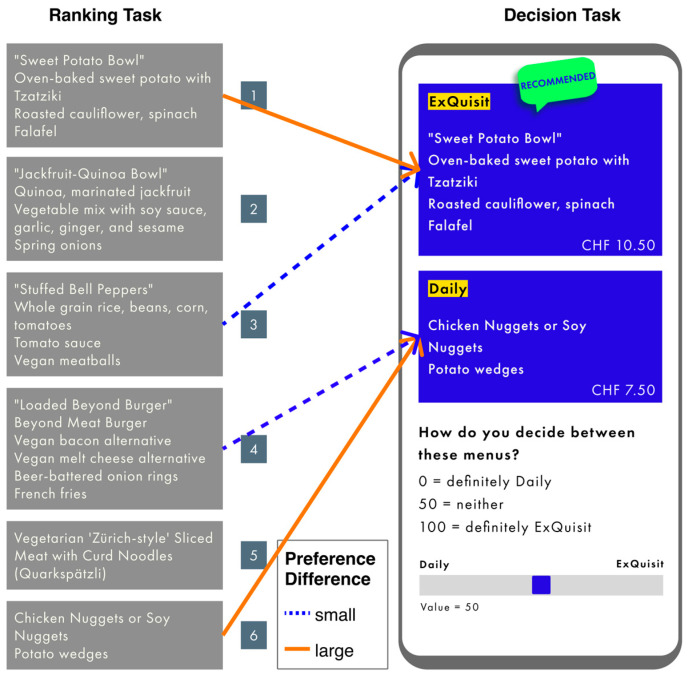
Stimulus material translated from German to English. Blue arrows symbolize the meals chosen for the small preference difference, the orange arrows for the large preference difference.

Stimulus material, procedure, and design were similar to Study 1 (its implementation was also done via Unipark), with the following exceptions: First, before the choice task, a *ranking test* was added to determine participants’ personal preference. The participants were presented with six menu options, which they had to rank in order of preference (most preferred at the top, least preferred at the bottom). The meals, all of which offered a vegetarian or vegan option, were displayed without a price and in random order (see Figure 7). Second, in the subsequent choice task, there were two conditions (large, small) regarding the preference difference, to which the participants were randomly assigned. Participants in the large preference difference condition were presented with their most and least preferred menu. Participants in the small preference difference condition were presented with the two menus they had ranked in the middle (Positions 3 and 4). Third, the menu choice was not recorded in binary form but on a metric scale ranging from 0 to 100 (0 = daily selection; 100 = exquisite selection). Fourth, since we found no position effects, we only manipulated price (again, cheap and expensive). Fifth, regarding the factors important for meal selection in the cafeteria, the following factors were added: queue length, allergies, labels, and an “other” option. Finally, the interpretation of the banner (nudge or marketing strategy) was not recorded using qualitative data but using a Likert scale ranging from 1 (perception as “nudge”) to 9 (perception as “marketing strategy”).

### 3.2. Results

Twenty-one participants who took less than 1.5 or more than 20 min were removed from the analysis (2.2% of the total). All analyses were conducted with R version 4.2.3 (15 March 2023).

**Figure 8:**
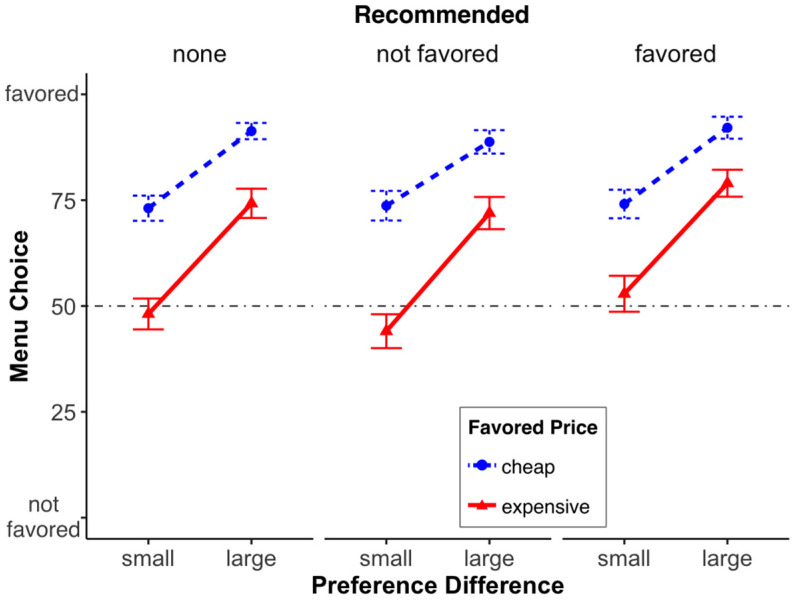
Menu choice (0 = not favored, 100 = favored) for the different factor combinations (recommended, preference difference, favored price). Error bars denote the standard error of the means.

**Figure 9:**
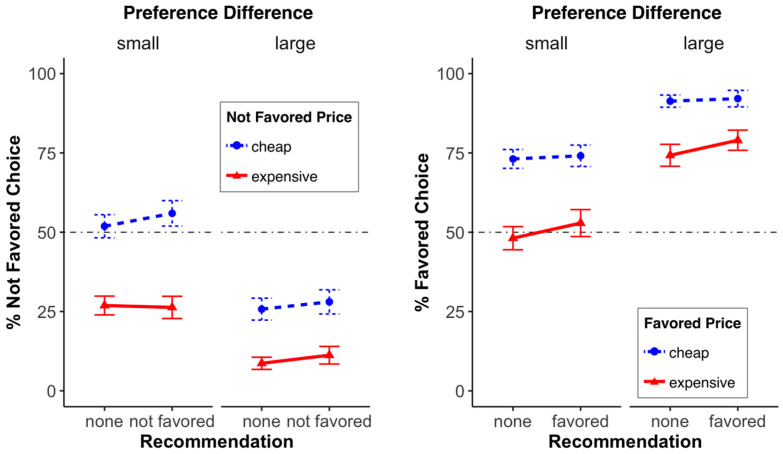
Impact of the recommendation, preference difference, and price on the choice of the least-favored meal (**left**) and the most-favored meal (**right**). The error bars represent the standard error of the mean.

*Menu Choice*. We conducted a 3 (menu recommendation: none, non-preferred, preferred) × 2 (price of the favored menu or simply “price”: cheap, expensive) × 2 (preference difference: small, large) between-subjects ANOVA over menu choice (scale 0 to 100). We found—as in Study 1—a significant main effect of price, *F*(1, 916) = 112.90, *p* < 0.001, with the cheap meal being chosen more often. Furthermore, we also found a significant main effect of preference difference, *F*(1, 916) = 128.90, *p* < 0.001, with a more pronounced difference between the choice of the preferred and non-preferred meal when the “preference difference” was large. We also found significant preference difference × price interaction, *F*(1, 916) = 6.18, *p* = 0.013; that is, the price effect was more pronounced when the difference in preference was small. All other interactions, 2-way or 3-way were not significant (all *F*’s ≤ 2.12; all *p*’s ≥ 0.12; see Figure 8 or Figure 9; both display the same data, but in a different way).

**Figure 10:**
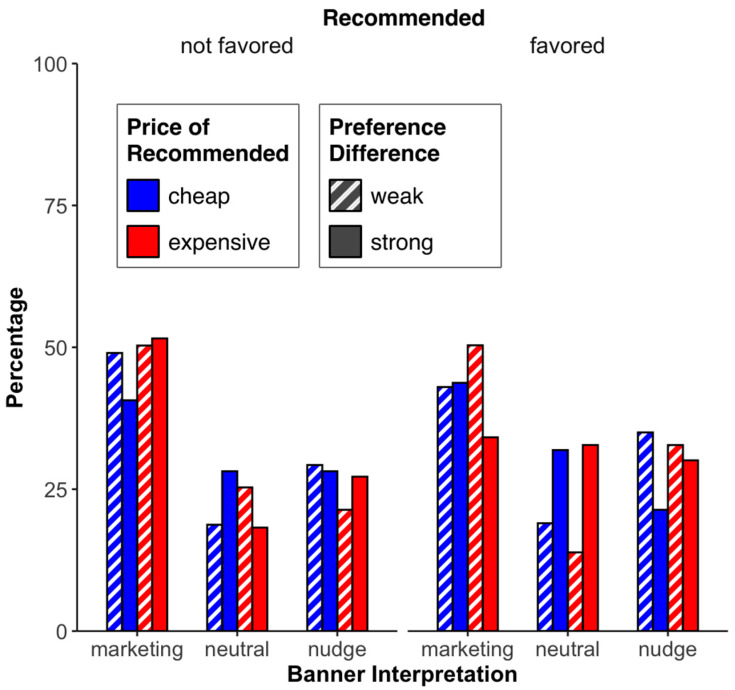
Percentage of the different banner interpretations for the different factor combinations (price of recommended, preference difference, recommended).

*Banner interpretation*. We conducted a 2 (menu recommendation: favored, not favored (participants from the «none» recommendation condition were excluded from this analysis)) × 2 (price of the recommended menu: cheap, expensive) × 2 (preference difference: small, large) ANOVA over the banner interpretation (scale from 1 = nudge to 9 = marketing). Unlike Study 1, none of the effects were significant (all *F*’s ≤ 1.80; all *p*’s ≥ 0.31; see Figure 10).

**Figure 11:**
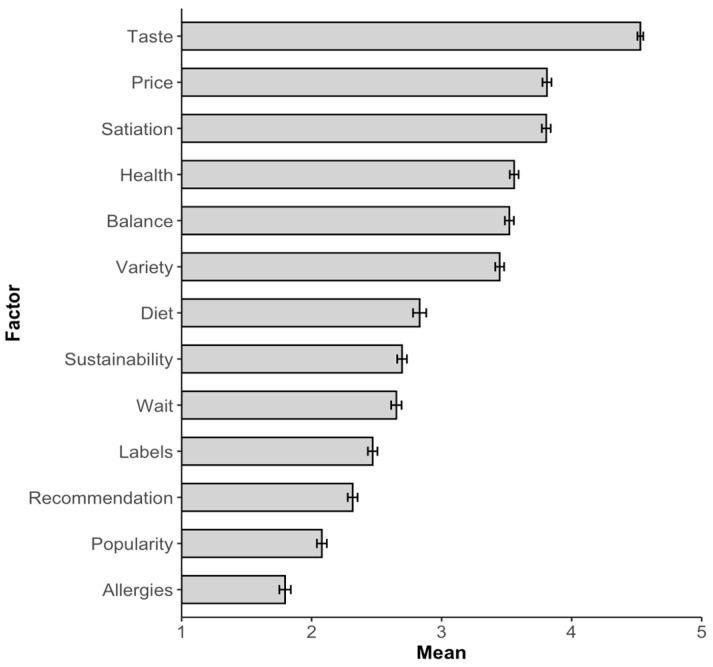
The relative importance of various factors influencing participants’ meal choices in the cafeteria (1 = not at all important to 5 = very important). The error bars represent the standard error.

*Menu choice relevant factors.* The order of importance of the factors for meal choice is similar to that in Study 1 (see Figure 11). Interestingly, although not considered one of the most important factors, *popularity* has a major influence on meal choice (see Study 1 and the discussion of Zanstra et al. [41]).

### 3.3. Discussion

To begin with, Study 1 is similar to the “small” preference difference condition of Study 2; both times the more popular meal (curry) or the preferred meal is chosen by about 66 or 61% of the participants and the less popular meal (dal) or the non-preferred meal is chosen by about 34 or 39% of the participants. However, while we found an interaction effect of recommendation (with price) in Study 1, no such effect was found in Study 2 (see the General Discussion section below for an explanation of the presence/absence of the effect). Similar to Study 1, Study 2 showed an effect of price on meal choice. There was also an effect of popularity or preference difference on meal choice. In addition, the difference in preference interacts with the price with a greater difference between the choice of the cheap and the expensive meal if the difference in preference is small.

## 4. General Discussion

Overall, we were interested in the effect of recommendations (from “dish of the day” to “chef’s recommendation”), which are used to change the framing of menu options [45], which in turn influences the consumer’s search process and, thus, their choice. In Study 1, we observed an effect of the *recommendation* on the more popular menu option (see also [21] for similar results and the classification of our label as an informative one), essentially boosting the sales of the cheap curry. The recommendation may have worked for the cheap menu, as it was interpreted as a good choice rather than one motivated by the restaurant managers to increase their profits. However, no effects of recommendation could be observed in Study 2. Instead, the observed price effect appears to have been influenced by the preference difference. The greater the difference in preference, the smaller the price difference.

Before discussing a possible reason for the presence or absence of the recommendation effect, we would like to point towards the findings of a number of studies, which also examined recommendations, but in a broader context, such as in a *larger choice set*. In these studies, mixed effects were also found in relation to the success of recommendations [46]. However, it should be noted that the main aim of these studies was to investigate how to promote the choice of vegetarian options. Accordingly, the remaining options in these studies are almost always exclusively non-vegetarian options. This contrasts with our two studies, where a vegetarian could always choose both menu options. When looking at these studies, Saulais et al. (2019) [45], for example, not only found an effect of the recommendation, but also that this effect was greater the larger the choice set (one vegetarian dish among one or two meat dishes; relative increase of 73% versus 129%). However, Bacon and Krpan (2018) could not confirm these results [47]; they found no effect of the recommendation in a relatively large choice set of one vegetarian meal among seven non-vegetarian meals (see also [48], for no effect on recommendations). However, when Bacon and Krpan (2018) only considered the frequent eaters of vegetarian dishes, a significant effect of recommendation could be observed. This can probably be explained by the fact that the recommendation made the vegetarian dish more salient, which led to it being noticed more often and, therefore, also chosen more frequently. Back to the Saulais et al. study, what is interesting for us is their condition with a choice with only two options (one vegetarian option and one meat option). In that condition, the recommendation had a greater effect when the recommendation was placed on the less popular menu option (see also [49], for similar results), namely the vegetarian option (which was chosen by 34% of participants in the no-recommendation control condition). In other words, the recommendation increased the choice of vegetarian dish by 73% in relative terms (only 11% for the more popular menu). While we found no effect for the less popular meal in Study 1, the authors’ findings for the more popular meal are consistent with those of our Study 1. However, the study by Saulais et al. (2019) has some complications. While the menu options were arranged horizontally in the control group, they were arranged vertically in the group with the recommendation. As can be seen from the position literature, this can lead to different results (see [28]). It is, therefore, unclear what the cause is (whether order or recommendation) for the recommendation effect observed in the study by Saulais et al. (2019). Last but not least, *price* (in addition to position) was never systematically manipulated in any of the studies mentioned.

Speaking of price: We hypothesize that the effect of the recommendation only becomes visible if the difference in the choice between the cheap and the expensive meals in the *control* condition is *small* enough. Accordingly, we find an effect in Study 1, where the difference—regardless of the meal (dal, curry)—is small. In Study 2, on the other hand, the difference (in all four control conditions) is too large for a visible effect of the recommendation. However, we can only speculate as to why the difference in choice (between a cheap and expensive meal) in the control conditions was sometimes small (in Study 1) and sometimes not (in Study 2). As you may have noticed, in Study 1, the menu options contained additional labels (e.g., sustainable, vegan). These labels may have caused participants to focus on them, reducing the importance of price, even though the labels were the same on both meals. However, this assumption needs to be tested in future experiments. Irrespective of our hypothesis of the effect of the recommendation, we see in Study 2 that the price effect depends on whether the difference in preference is small or large. The greater the difference, that is, the further one moves in the direction of either extreme (completely favored or completely not favored) the less pronounced the price effect becomes. A similar effect can be observed in elections (on personal or factual issues). Citizens who are rather indifferent in voting issues, for example, are more likely to be manipulated in their voting behavior than citizens with a strong bias (no matter in which direction; one-sided; see [50]).

Before ending the discussion, we would like to address some potential limitations of our study. For example, it could be argued that popularity and personal preference do not measure the same thing and that Study 1 is not about popularity but about familiarity (i.e., Swiss people’s familiarity with curry dishes compared to dal dishes), which is different from personal preference. However, we reject this objection not only on conceptual grounds (see, for example, [49], who also equated familiarity with popularity), but also based on the data. What do we mean by this? We have re-analyzed the data in Study 2, but only with the data and participants for whom the favored menu was chosen just as often as the curry in Study 1. The pattern found was identical to that found in Study 2 with all data. Another limitation could be that in Study 1 menu choice was measured with a binary measure, whereas in Study 2, it was measured with a continuous scale (0–100). Due to the study by Dolnicara and Gruen (2007) [51], who found no difference in the response format used (binary or metric), we do not expect this to cause different results. Another potential limitation in our study is that it is based on responses from students only. Factors such as price sensitivity or the reaction to advertising campaigns can have very different effects in different socio-demographic groups [52,53,54]; (see also the topic of consumption of PBMSs in the introduction), and the same could also apply to the effect of nudges such as recommendations or positions. Therefore, the inclusion of several subgroups in future studies could provide deeper insights.

In summary, while we have discussed various reasons why recommendations might not have had an effect, our results align with the self-reported factors that influence menu choices, where recommendations are consistently low on the list. Although such self-reported measures do not always align with reality, it is possible that, at least when choosing from a cafeteria menu, we might not be as easily influenced as the nudging literature suggests. While this implies that recommendations may not be an effective tool for nudging people toward healthier or more sustainable choices, it also suggests that recommendations cannot be easily exploited for blatant marketing tactics. However, further research, particularly in different contexts, is needed to definitively remove recommendations from the list of successful nudging interventions.

## 5. Conclusions

In recent years, digitalization has changed our daily food choices, especially in the hospitality industry. This process has been accelerated by the COVID pandemic, especially in an effort to address customers’ concerns about health and safety [55]. QR codes for contactless food ordering in restaurants or online food ordering platforms such as Uber Eats have become commonplace in our daily routines.

This change has also opened the door for the use of digital nudges (for a definition, see [56], or [57]). However, digital nudges are now mainly researched in areas such as privacy/security, e-commerce/marketing, and social media [58], but not in online food choices [59,60]. However, since “nudging in the digital realm differs from its analog counterpart in important ways” [58] (p. 1), this is a serious shortcoming. One of the key differences mentioned is the possibility of personalization in digital nudging (also see [59,61]).

It is precisely on this topic that our work has tried to shed more light, including in the matter of personalization (which is implemented as “personal preference”). Hence, our research findings can provide valuable insights for researchers and practitioners who use digital platforms to promote healthier and more sustainable food choices.

## Data Availability

The original contributions presented in the study are included in the article, further inquiries can be directed to the corresponding author.
